# Should the Health Community Promote Smokeless Tobacco (Snus) as a Harm Reduction Measure?

**DOI:** 10.1371/journal.pmed.0040185

**Published:** 2007-07-03

**Authors:** Coral E Gartner, Wayne D Hall, Simon Chapman, Becky Freeman

## Abstract

Background to the debate: The tobacco control community is divided on whether or not to inform the public that using oral, smokeless tobacco (Swedish snus) is less hazardous to health than smoking tobacco. Proponents of “harm reduction” point to the Swedish experience. Snus seems to be widely used as an alternative to cigarettes in Sweden, say these proponents, contributing to the low overall prevalence of smoking and smoking-related disease. Harm reduction proponents thus argue that the health community should actively inform inveterate cigarette smokers of the benefits of switching to snus. However, critics of harm reduction say that snus has its own risks, that no form of tobacco should ever be promoted, and that Sweden's experience is likely to be specific to that culture and not transferable to other settings. Critics also remain deeply suspicious that the tobacco industry will use snus marketing as a “gateway” to promote cigarettes. In the interests of promoting debate, the authors (who are collaborators on a research project on the future of tobacco control) have agreed to outline the strongest arguments for and against promoting Swedish snus as a form of harm reduction.

## Coral Gartner and Wayne Hall's Viewpoint: Smokers Who Switch to Snus Reduce Their Health Risks

Over the past 40 years, high taxes on cigarettes, advertising bans, and restrictions on smoking have almost halved adult rates of cigarette smoking in Australia and the United States [1,2]. Nonetheless, around one in five adults in these countries still smokes tobacco because they are either unable or unwilling to stop.

We think it would be good public health policy to encourage inveterate smokers to adopt less harmful ways of using nicotine, to reduce the disease burden caused by tobacco smoking [3,4]. This policy—labelled tobacco harm reduction (THR)—has prompted heated debate within the tobacco control community.

Critics of THR point to the failure of “light” cigarettes to reduce health risks because of compensatory smoking (e.g., blocking ventilation holes, inhaling more intensely, smoking each cigarette down to a smaller butt length, and smoking a greater number of cigarettes). Similar strong doubts surround the safety of any new combustible tobacco products, given that it takes 40 to 50 years to evaluate their potential harms or benefits [3].

Low nitrosamine oral snuff, or Swedish snus, is not vulnerable to the criticisms levelled at light cigarettes. Snus has been used by substantial proportions of nicotine consumers in Sweden over the past several decades, which has allowed its effects on smoking prevalence and health to be studied.

Similar to other types of smokeless tobacco products, snus may increase the risk of some cancers [5,6,7], and there may be some risk of cardiovascular disease caused by nicotine. But its cardiovascular risks are certainly lower than those of smoking, it has no respiratory risks, and its oral cancer risk is probably much lower than that of conventional chewed tobacco because snus has a much lower nitrosamine content [8,9]. Studies in Sweden, where men have used snus for 20 years, have so far failed to detect any increase in oral cancer or cardiovascular disease rates [5,8,10]. On current evidence the health risks of snus are comparable to those of regular alcohol use rather than cigarette smoking.

### Impact on Tobacco Use by Youth

Many critics are understandably concerned that the tobacco industry will use snus to promote nicotine use among adolescents and young adults. This is a possibility that should be addressed by tighter regulation of all existing tobacco products [3]. For example, snus (and other tobacco products, including cigarettes) could be made less desirable to new users by: prohibiting flavouring additives, regulating packaging and labelling (e.g., generic packaging), and prohibiting all tobacco advertising.

### Aggregate Effects on Public Health

Critics of snus argue that its promotion may reduce overall tobacco-related disease in current smokers at the cost of increasing tobacco use in the population. Whether snus produces net aggregate harm or benefit will depend on who in the population uses it. If its use is confined to current smokers (who would not have otherwise quit tobacco), then snus would clearly produce a net benefit, as it appears to have done in Sweden.

Snus would produce net harm only if: (1) it had no effect on current rates of smoking; and (2) it was used in the vast majority of cases by non-smokers and former smokers [11]. Neither case has been observed in Sweden, where smoking prevalence has declined because most snus users were formerly smokers [12]. Moreover, substantial health gains have been achieved in the face of a relatively high prevalence of snus use (21% of men are daily snus users). The Swedish population prevalence of tobacco use has remained relatively steady at around 40% but the proportion of users who smoke has gradually declined, with snus now accounting for 58% of daily tobacco use [13]. Despite this high prevalence of snus use, tobacco-related mortality in Sweden is among the lowest in the developed world [14]. If the goal of tobacco control is to reduce tobacco-related disease, rather than tobacco use per se, then the promotion of snus use by inveterate smokers is a promising public health policy.

### Effects on Other Tobacco Control Policies

Critics also contend that increased snus use would reduce smoking cessation because smokers who would otherwise quit due to the inconvenience of smoking bans will use snus when smoking is not allowed and smoke when smoking is allowed.

This criticism confuses the primary purpose of smoking bans, which is to protect non-smokers from the harmful effects of second-hand smoke, with the secondary benefit of increasing smoking cessation. In any case, increased snus use in Sweden did not impede smoking cessation efforts. In fact, smoking prevalence and tobacco-related mortality have both declined in Sweden as snus use has increased. This trend appears to be because people who start using snus are much less likely to start smoking than those who have never used snus, while smokers who start using snus have a higher smoking cessation rate than smokers who do not [13].

### Public Health Paternalism

Smokers have an ethical right to be accurately informed by public health officials about THR products. It is paternalistic to deny smokers this information for fear that population nicotine use may increase. As Kozlowski [15] has argued, informing smokers about THR is an effective public health measure that properly respects their autonomy. The failure to do so, or worse, the dissemination of misinformation that snus and smoked tobacco are equally harmful, is untruthful and risks creating public mistrust of health messages about tobacco use [16,17].

### Conclusion

Tobacco smokers who switch to snus will reduce the risks of their tobacco use. Based on the Swedish experience, there is a strong prima facie case on public health and ethical grounds for recommending snus to inveterate smokers who want to reduce their health risks and for considering public policies (such as lower taxes for snus and public information campaigns) to promote its use by smokers. The legitimate concerns of THR opponents will be addressed by better regulation of *all* tobacco products rather than either bans on snus (as in Europe and Australia) or misinformation about the health risks of snus (as in the US where its sale is legal). Public health and tobacco control professionals should accordingly work for better regulations to make all tobacco products less attractive to new (and existing) users rather than attempting to discourage smokers from switching to snus through misleading claims that snus use is as risky as cigarette smoking.

## Simon Chapman and Becky Freeman's Viewpoint: There Are Five Reasons to Be Cautious about Snus

The Swedish snus experience deserves the close attention of all concerned with reducing the burden of disease caused by tobacco use. There has been much acrimony in the debate about the international implications of Sweden's dramatic reduction of its burden of disease caused by tobacco. This acrimony reflects clashes of differing ideological values aroused by the harm reduction principle. Harm reduction's core principle is that risk reduction strategies can complement risk elimination (i.e., cessation) strategies in reducing the net harm from tobacco use in populations. This principle is heretical to absolutists intolerant of any form of tobacco use.

Reviews of the Swedish experience have raised few doubts that its unprecedented decline in tobacco-caused mortality is associated with the rise of snus use at the expense of smoking [3,8]. Smokers have switched to snus and cohorts of young people who would have likely taken up smoking have instead adopted snus [18,19]. Significant numbers also use snus as a means of stopping tobacco use altogether [13].

But unbridled enthusiasts for the Swedish experience to be disseminated globally nonetheless face five challenging questions: (1) Is Sweden's snus experience shaped by culturally specific factors that may not transfer to other nations? (2) How would the benefits of snus be communicated to potential users, and would tobacco companies use snus advertising to subvert global advertising bans? (3) Because the tobacco industry aims to promote dual use (smoking and snus), how do snus enthusiasts defend against arguments that such use would increase rather than decrease harm? (4) What is the marginal cost utility of the tobacco control community putting its effort into informing the public about snus compared with the gains to be made by continuing “orthodox” tobacco control? (5) Why is the public health community's energy currently being invested in supporting tobacco company efforts to freely market snus, instead of putting this energy into efforts to promote high yielding pharmaceutical “clean” nicotine?

### Culturally Unique?

While there are legislative barriers to the sale of snus in Europe, Australia, New Zealand, and several other nations, none exist in most nations. Why then has snus failed to take off elsewhere in the way it has taken off in Sweden? Global marketing has seen formerly culturally unique products and practices succeed internationally, but many fail. Snus users typically hold a pinch of loose snus or a snus “portion” in their mouths for around 13–15 hours a day. This is hardly a minor, occasional behaviour but one requiring a major commitment. Smokeless tobacco sales are growing in the US [20] but seemingly nowhere else. Smokeless tobacco has failed to become established in any nation other than those with long-standing cultural traditions of use. This failure suggests widespread smokeless tobacco use is culture-bound and highly resistant to migration.

### Communicating Benefits

There would be little point in making snus available if its benefits could not be actively communicated to potential users. Tobacco companies face a global shut-down of advertising and promotion under the Framework Convention on Tobacco Control and have already begun using harm reduction to argue for the retention of advertising [21]. Given the tobacco industry's long history of mendacity, we can be certain that snus advertising will be used for the wider benefit of the industry. For as long as both cigarettes and smokeless products are marketed by the same companies, collateral benefits to be obtained from riding the harm reduction moral imperative (such as dual use—see below) will be foremost in cynical industry plans.

### Dual Use

Snus enthusiasts in the public health community focus on the potential of snus to take people away from smoking. However, transnational tobacco companies are already marketing snus using slogans that mention smoking, such as: “When you can't smoke, snus”. Here, there is no suggestion of stopping smoking, but of use of *both* cigarettes and snus. Smoking bans driven by the evidence on second-hand smoke have caused wholesale smoking cessation and reduced cigarette use [22]. The tobacco industry is desperate to stem this loss of tobacco sales. Promoting dual cigarette/snus use is therefore harm *increasing*, not reducing. Forecasts of increased snus use leading to reduced smoking in populations therefore need adjusting to incorporate tobacco industry efforts to promote dual use.

### How Much Effort Should We Put into Harm Reduction?

In nations with robust, comprehensive tobacco control programs, smoking rates continue to fall, with weak evidence for “hardening” of the smoking population. (“Hardening” means that the remaining smokers are mostly “hard core”, heavily addicted smokers who are unwilling or unable to quit [23].) In fact, there is often a “softening” effect with quit rates remaining high and daily smoking frequency reducing [24]. In Canada, only 13.4% of the population now smokes daily, with another 4.2% smoking less than daily [25]. Youth smoking rates are at an historic low in Australia [26]. Tobacco control operates on limited budgets and resources. How much of these resources should be quarantined to promote snus, given that companies could not be trusted to do it? We need cost–benefit comparisons to compare the effects of orthodox tobacco control with the effects of promoting snus.

### High-Dose “Clean” Nicotine

Real world use of current generation pharmaceutical nicotine (nicotine replacement therapy) has not lived up to the promise of many clinical trials [27]. Limited but promising evidence now available suggests that high-dose pharmaceutical nicotine is more effective in reducing smoking [28]. High-dose pharmaceutical nicotine would not be marketed by tobacco companies. Instead, it would be subject to regulatory control that would prevent the dual-use promotional abuses discussed above through under-the-counter sales at pharmacies. Again, issues of cultural transferability apply. Would smokers view use of high-dose nicotine replacement therapy as an acceptable substitute for the social rituals of smoking? The relative lack of advocacy for potent “clean” nicotine delivery is intriguing and is rumoured to have much to do with internal pharmaceutical industry concern about nicotine being less benign than previously thought. Nicotine's role in apoptosis is a fertile area for further research. The possible health effects of nicotine must surely be considered in any proposal that would encourage widespread use of snus, which delivers significant levels of nicotine [29].

## Coral Gartner and Wayne Hall's Response to Simon Chapman and Becky Freedman's Viewpoint

The health community should beware of over-estimating the potential public health impact of snus. Snus use may not fully replace cigarette smoking but it may hasten its demise, and that would be a substantial public health gain.

In Australia, a growing smokeless tobacco market was curtailed by government bans in the 980s following increased uptake in response to promotional activities [30]. This growing market indicates that some Australian smokers were willing to try smokeless tobacco. Bans on sales of smokeless tobacco products in Australia and Europe are a major impediment to finding out if snus use is a culture-bound practice and whether bans are justified.

Promotion of snus does not preclude the promotion of other high-dose, “cleaner” nicotine products. Indeed, we would support steps to make both more available. In most countries, the only clean forms of nicotine available are low-dose medicinal replacement products that are unattractive to social users. Unfortunately, higher-dose recreational nicotine products will encounter substantial difficulties in gaining access to new markets because of irrational regulation that is more stringent for pharmaceutical nicotine and snus than for smoked tobacco. As a recreational tobacco product, snus may seem to smokers like a more natural substitute for cigarettes than clean nicotine preparations.

Diversion of funding and human resources from other tobacco control strategies to snus promotion is certainly a concern. But so too is the effort invested by some in the tobacco control community to actively opposing snus use and misleadingly claiming that snus use is as harmful as tobacco smoking. This energy could be better invested in arguing for better regulation of all tobacco products and for lower taxes on snus and clean nicotine products to enable them to more successfully compete with cigarettes among current tobacco users.

## Simon Chapman and Becky Freeman's Response to Coral Gartner and Wayne Hall's Viewpoint

Gartner and Hall speak of the virtues of inveterate smokers switching to snus, implying that there is an intransigent hard core of smokers. The tobacco industry also routinely suggests the existence of such a “hard core”. Yet the size of this group is constantly shrinking. Quit rates in nations with comprehensive tobacco control programs are accelerating, and not declining. So whose interests will be served by diverting potential quitters into snus, particularly when tobacco marketing is already explicitly using snus promotion to promote cigarettes?

We grant that indoor smoking bans were premised on reducing harm to non-smokers. But such bans have had enormous collateral benefits for smokers themselves, by stimulating them to quit. Indeed smoking bans have been widely appreciated by a large majority of smokers. The industry's “dual use” ambitions—that is, using snus to promote cigarettes—could destroy much of these collateral benefits. Again, whose interests would be served by subverting the beneficial effect of smoking bans?

We note that Gartner and Hall call for a regulatory scheme to safeguard against the snus industry from appealing specifically to teens by using flavouring, alluring packaging, and teen marketing. But such schemes may take decades to implement, particularly when industry is likely to resist such regulations. While these are being negotiated, smoking prevalence will continue to fall. In nations with high smoking prevalence, the argument for snus is more compelling, but there is nothing preventing it being marketed now in, for example, China. Yet smokeless tobacco has never taken off in any nation without a smokeless cultural tradition.

So we reiterate: smokeless tobacco has low appeal for the overwhelming majority of the world's smokers. There are profound risks in letting tobacco industry tigers off their leash to use snus to subvert the hard-won provisions of the Framework Convention on Tobacco Control—provisions that include a ban on all tobacco advertising. Such a ban has already been achieved in some nations, but not in the US, from where much of the enthusiasm for snus now comes.

## Abbreviations

THR: tobacco harm reduction
